# On the Function of Trans-Splicing: No Evidence for Widespread Proteome Diversification in Trypanosomes

**DOI:** 10.1093/gbe/evz217

**Published:** 2019-10-10

**Authors:** Cameron M Soulette, Oliver Oliverio, Scott W Roy

**Affiliations:** 1 Department of Biology, San Francisco State University; 2 Quantitative Systems Biology, University of California, Merced; 3 Molecular, Cellular & Developmental Biology, University of California, Santa Cruz, Santa Cruz, CA

**Keywords:** molecular evolution, spliced leader, trans-splicing, trypanosome, comparative genomics, bioinformatics

## Abstract

A long-standing mystery of genomic/transcriptomic structure involves spliced leader trans-splicing (SLTS), in which short RNA “tags” transcribed from a distinct genomic locus is added near the 5′ end of RNA transcripts by the spliceosome. SLTS has been observed in diverse eukaryotes in a phylogenetic pattern implying recurrent independent evolution. This striking convergence suggests important functions for SLTS, however no general novel function is known. Recent findings of frequent alternative SLTS (ALT-TS) suggest that ALT-TS could impart widespread functionality. Here, we tested the hypothesis that ALT-TS diversifies proteomes by comparing splicing patterns in orthologous genes between two deeply diverged trypanosome parasites. We also tested proteome diversification functions of ALT-TS by utilizing ribosome profiling sequence data. Finally, we investigated ALT-TS as a mechanism to regulate the expression of unproductive transcripts. Although our results indicate the functional importance of some cases of trans-splicing, we find no evidence for the hypothesis that proteome diversification is a general function of trans-splicing.

The disparity between gene number and organismal complexity across organisms raises an interesting question of how increased complexity is produced with a relative economy of genes (e.g., Lander et al. 2001). One mechanism that may help explain this disparity is alternative splicing, in which a single gene can give rise to distinct mRNA transcripts that code for different primary polypeptides (Maniatis and Tasic 2002; Modrek and Lee 2002). However, proteome diversification via alternative splicing appears to be rare in nearly all eukaryotic lineages (Sorek et al. 2004; McGuire et al. 2008), suggesting that alternative splicing is neither a general nor sufficient explanation.

Recent transcriptome-level analyses suggest that a general role in proteome diversification could be played by spliced leader trans-splicing (SLTS), a process by which a short RNA sequence called a spliced leader (SL) is spliced to protein-coding pre-mRNAs by a spliceosomal reaction (Sutton and Boothroyd 1986). In an elegant study utilizing a high-throughput RNA-seq method to identify SLTS splice sites genome-wide, Nilsson et al. (2010) authors report widespread “alternative trans-splicing” (ALT-TS) in *Trypanosoma brucei*, in which SLs are alternatively spliced to alternative sites within a gene’s transcripts, leading to transcript isoforms with distinct 5′ ends (fig. 1*a*). Consistent with a functional role, many ALT-TS events showed differences in splicing across different *T.brucei* developmental stages.

**Fig. 1.**
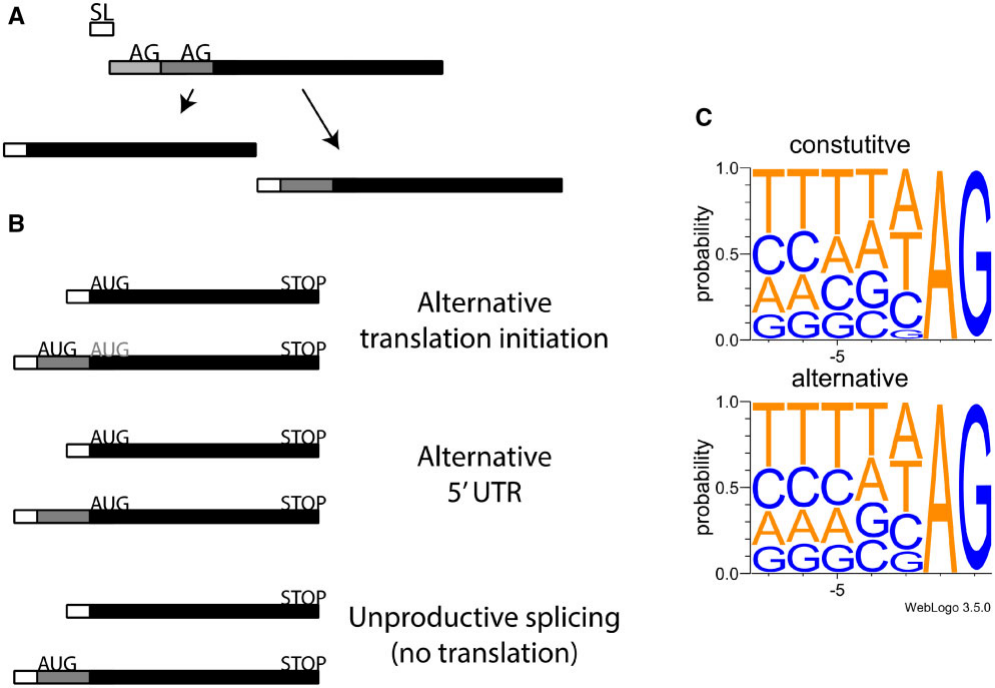
—Alternative spliced leader trans-splicing occurs through the addition of the spliced leaders at distinct acceptor sites of pre-mRNA derived from the same gene locus. (*A*) mRNAs that undergo spliced leader addition at more than one site give rise to mRNAs with distinct 5′ ends. (*B*) Possible functional outcomes of alternative spliced leader trans-splicing. (*C*) Nucleotide context of acceptor sites used by spliced leader trans-splicing, constitutive sites (top panel), and alternative sites (bottom).

Interestingly, many alternative SLTS sites lie downstream of the 5′ end of the genic ORF, suggesting that translation initiation could occur at different sites on different transcript isoforms, leading to production of alternative protein isoforms. Indeed, follow-up molecular studies in the parasite *Trypanosoma cruzi* showed that different trans-splicing isoforms of the isoleucyl-tRNA synthetase gene encode multiple protein isoforms through the production of ALT-TS products with distinct 5′ ends (Rettig et al. 2012). The authors hypothesize that proteome diversification might represent an important general functional role for ALT-TS. This possibility is particularly exciting for two reasons. First, observed phylogenetic distributions suggests that SLTS may preferentially evolve in multicellular animals, consistent with a role in the evolution of organismal complexity (Modrek and Lee 2002; Irimia et al. 2007; Kim et al. 2007). Second, SLTS is found in several diverse lineages of parasites (Lukeš et al. 2009; Roy and Irimia 2009; Douris et al. 2010), thus SLTS may provide novel intervention targets. To test the possibility that ALT-TS often confers important functions in proteome diversification, we performed a comparative genomic analysis by leveraging transcriptomic and genomic data for a wide variety of trypanosome parasites.

## Results and Discussion

### Trans-Splicing in T. brucei

We first identified ALT-TS genes in *T.brucei* by utilizing available *S*pliced *L*eader *T*rapping (SLT) RNA-seq data sets (GEO accession GSE22571). SLT is a high-throughput method for identifying the 5′ end of trans-spliced mRNAs (Nilsson et al. 2010). We aligned available SLT data to the *T.brucei* genome using Bowtie (Langmead et al. 2009) to identify STLS 3′ acceptor sites. We then assigned each SLTS site to the closest *T.* *brucei* gene on the same strand (see Materials and Methods). We identified genes in which ALT-TS was supported by substantial overall SLT read counts (≥3 total SLT reads supporting each of ≥2 SLTS sites) and proportions (≥2 SLTS sites with ≥10% of a gene’s most supported SLTS site).

To explore mechanisms of ALT-TS in *T. brucei*, we compared acceptor splice sites between constitutive SLTS and ALT-TS splice sites. Constitutive and ALT-TS sites showed very similar consensus sequences (fig. 1*c*), as did comparisons between ALT-TS sites with different levels of usage (e.g., major vs. minor forms; data not shown), with the only clear difference being a slightly greater tolerance for ALT-TS sites of a guanine directly preceding the AG terminal dinucleotide (fig. 1*c* lower panel). These observations are consistent in some cases of alternative *cis*-splicing, in which splice sites for alternative splicing events show weaker adherence to splice site consensus motifs than do constitutive *cis*-splicing sites (Clark and Thanaraj 2002), as well as in a specific case in *Caenorhabditis elegans*, in which alternative acceptor sites often differ by the presence of a purine at this position (Ragle et al. 2015).

### ATG Conservation Does Not Support Conserved Alternative Protein Production

We next turned to the protein-coding implications of ALT-TS. Transcripts produced by splicing at internal ALT-TS sites within an open reading frame lack the putative 5′ translation initiation site, thus if such transcripts are translated, translation must be initiated from a downstream ATG. Such ALT-TS products would produce distinct N-terminally truncated protein isoforms (e.g., fig. 1*b*). Notably, our set of 2,597 *T. brucei* ALT-TS genes included 524 genes, with substantial open reading frame lengths (supplementary fig. 2, Supplementary Material online), for which well-supported ALT-TS sites lay upstream of different in-frame ATGs. These data are summarized in supplementary table 1.

**Fig. 2.**
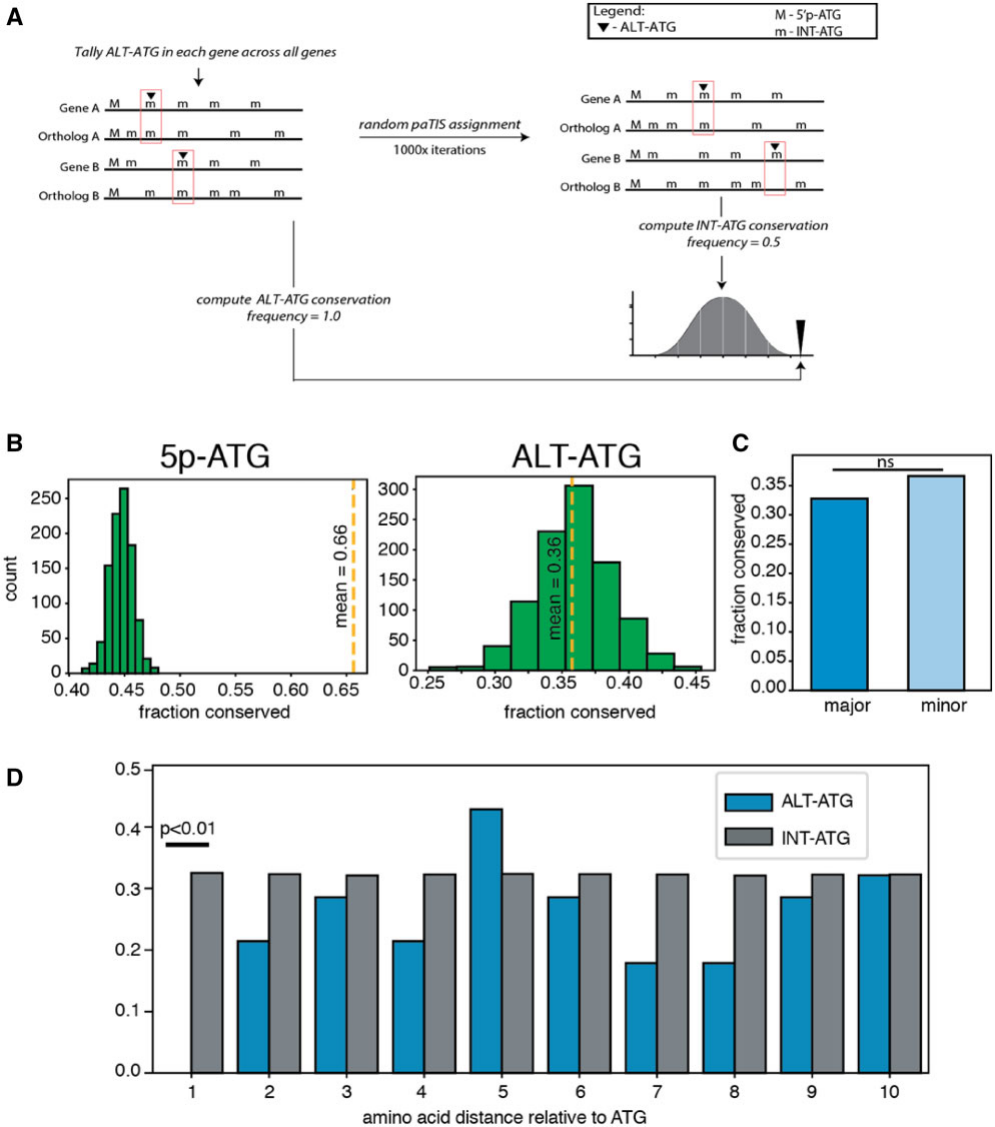
—Evolutionary conservation of ALT-ATGs is similar to INT-ATGs. (*A*) Diagram of permutation workflow setup for comparing ALT-ATG to background (INT-ATG). (*B*) Left panel—conservation of 5p-ATG represented as yellow dashed line. Null distribution (INT-ATG conservation represented as green bars). Right panel—same comparison as Left panel, but yellow dashed line represents conservation of ALT-ATGs. (*C*) Conservation comparison between major and minor ALT-ATGs. (*D*) Position specific conservation of ATGs near INT-ATGs and ALT-ATGs (blue bars).

We next sought to determine whether such ALT-TS-coupled internal ATG codons (“ALT-ATGs”) represent bona fide translation initiation sites. We reasoned that if ALT-ATGs initiate translation for functionally important alternative protein isoforms, then they should be preferentially evolutionarily conserved, specifically showing higher levels of conservation than other internal ATG codons (“INT-ATGs”). Moreover, if ALT-ATGs initiate translation, then they could even approach the level of conservation of the 5′ ATGs of ORFs, which are confident sites of translation initiation (“5p-ATGs”). Therefore, we compared levels of evolutionary conservation between *T. brucei* and *T. cruzi* for these three classes of in-frame ATG codons. Orthologous genes were defined by protein BLAST reciprocal best hit, and ATG conservation was measured using a permutation-based approach that accounts for gene specific conservation levels (see Materials and Methods; fig. 2*a*).

From our candidate list of 524 ALT-TS *T.brucei* genes, 430 has corresponding orthologs in *T.cruzi*. We first investigated the extent to which ALT-TS acceptor sites are conserved. We examined 574 acceptor sites internal to the main open reading frame, and found no substantial conservation enrichment between acceptor AG dinucleotides and random AG dinucleotides in the same reading frame (supplementary fig. 1*a*, Supplementary Material online). Next, we investigated the conservation of 5p-ATGs and ALT-ATGs. Consistent with the notion that translation initiation sites are preferentially conserved, we found that confident 5p-ATGs are much more highly conserved than are INT-ATGs (249/430; *P* < 0.01 fig. 2*b* left panel). By contrast, we found that ALT-ATGs were conserved at a level indistinguishable from INT-ATGs (172/430; *P* = 0.54). Notably, ALT-ATGs associated with more common STLS sites—those from more common transcript isoforms—did not show elevated conservation levels, consistent with a general lack of ALT-ATG conservation (*P* = 0.512 by χ^2^ two-tailed test) (fig. 2*c*). Given the >100 Myr divergence between *T.brucei* and *T.cruzi* (Stevens et al. 1999), this result could partially be explained by lineage-specific loss of ALT-ATG function. To rule out lineage-specific loss of ancestral ALT-ATG function, we tested for conservation of ALT-ATGs across other publicly available trypanosome transcriptome data sets (retrieved from TriTypDB). We found similar patterns of ATG conservation across various trypanosome species (supplementary fig. 1*b*, Supplementary Material online), in which 5p-ATGs are conserved a significantly higher rate than INT-ATGs, and ALT-ATGs are conserved at levels similar to INT-ATGs.

We next hypothesized that ALT-ATGs need not be conserved at the same exact amino acid position, and that ATG codons conserved in close proximity could serve as functional translation initiation sites. This hypothesis is consistent with the case of isolecucyl tRNA synthetase (Ile-tRS), in which an ATG not conserved at the same site, but in close proximity, serve to initiate translation of dual targeted Ile-tRS (Nilsson et al. 2010; Rettig et al. 2012). We therefore tested if the frequency of ATG codons in alignment positions near ALT-ATGs are conserved at a significantly higher rate than INT-ATGs. Our results showed no elevation of ATGs at alignment positions near ALT-ATGs (fig. 2*d*).

Results from our transcriptome-wide conservation analysis were inconsistent with ALT-TS patterns associated with isoleucyl Ile-tRS. To reconcile this incongruity, we hypothesized that perhaps ALT-ATG usage may be limited to genes localized to mitochondria, similar to the case of Ile-tRS (Rettig et al. 2012). We studied 16 genes encoding mitochondrially targeted products (retrieved from supplemental table 4 of Panigrahi et al. 2009). 69% (11/16) of ALT-ATGs in this subset of genes were conserved between species, comparable to the 68% found among INT-ATGs in these genes (*P *>* *0.05 by χ^2^ two-tailed test).

### Ribosomal Profiling Does Not Support Widespread Translation Initiation from ALT-ATGs

**Fig. 3.**
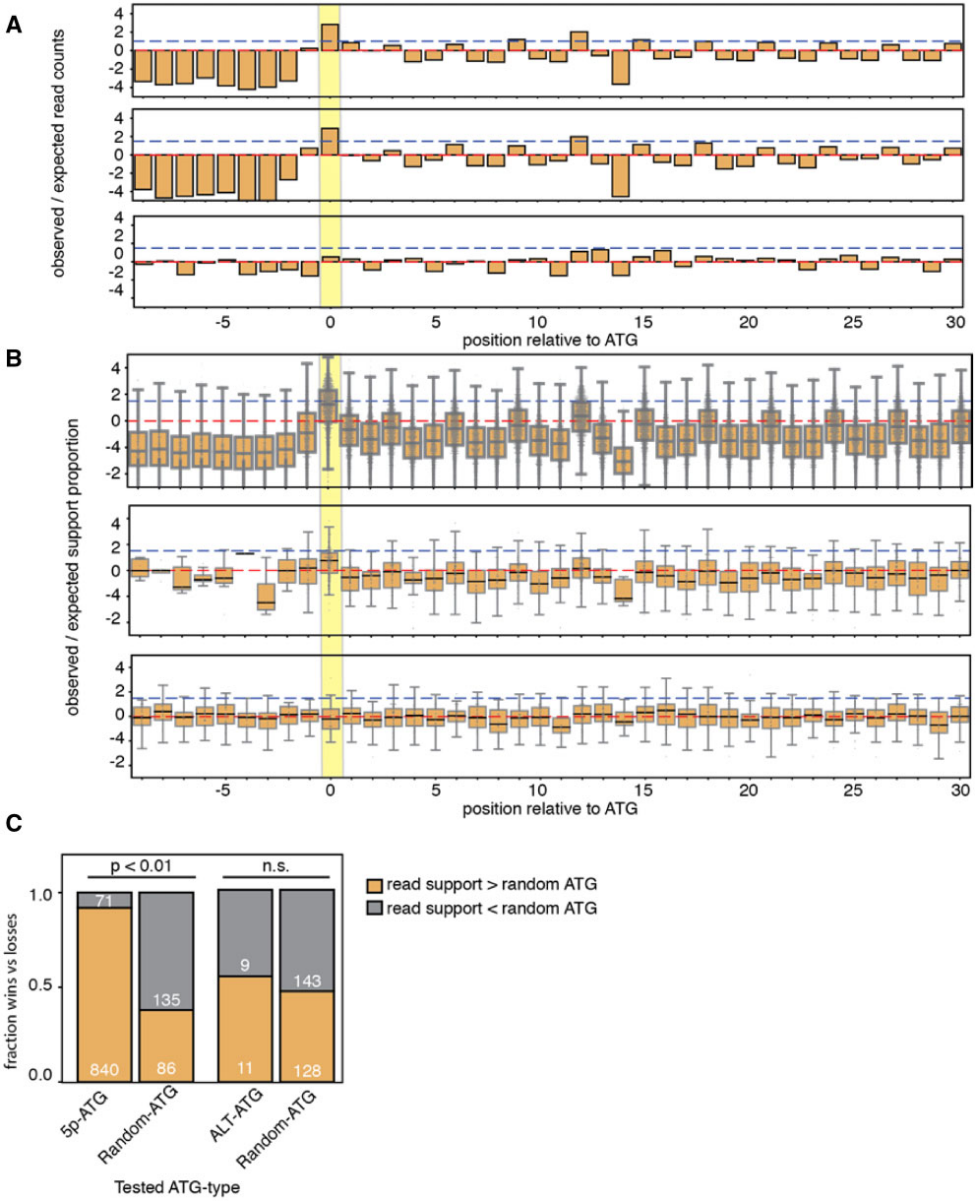
—ALT-ATGs do not share TIS signatures observed in ribosome profiling data. (*A*) Ribosome profiling summary read support distributions relative to 5p-ATGs from all genes (top panel), ALT-TS genes (middle), and ALT-ATGs (bottom panel). Red and blue dashed lines represent expected totals and log2-fold change of 1.5. Yellow bar highlights position of putative translation initiation site. (*B*) Proportion of ribosome-profiling nucleotide-specific support within 40-nt window of 5p-ATG for all genes (top panel), 5p-ATG for ALT-TS genes (middle panel), and ALT-ATGs (bottom panel). (*C*) Comparison of ribo-seq read support between different ATG types and randomly selected INT-ATG.

We next used ribosomal profiling data (ribo-seq) to test for evidence of translation initiation at ALT-ATGs. Ribo-seq is a high-throughput next generation RNA sequencing method to characterize mRNAs bound by actively translating ribosomes (Ingolia et al. 2009). We could test for translational efficiency of ALT-ATGs by identifying ribo-seq reads containing SL sequences that splice directly upstream from ALT-ATGs. However, given the fragment length and sequencing depth the ribo-seq data used in this study (Vasquez et al. 2014; PRJEB4801), we could only identify 10 ALT-TS genes with substantial read coverage (>20 reads), none of which contained read support for ALT-ATGs (data not shown). Alternatively, previous ribo-seq analyses have established that sequenced reads corresponding to the beginning of open reading frames (i.e., 5′ ends of ORFs) are significantly enriched in comparison to sequenced reads corresponding to the rest of the ORF, which is caused by cycloheximide pausing of ribosomes at translation initiation sites (Ingolia et al. 2009) (fig. 3*a* left panel). This read enrichment signature that defines open reading frames allows us to test whether our ALT-ATGs (defined without reference to direct data about translation) share this signature of translation initiation.

We tested for translation initiation signatures using *T.brucei* ribo-seq data (Vasquez et al. 2014). We aligned ribo-seq data to the *T.brucei* transcriptome with bowtie2 using the –local-sensitive option (Langmead et al. 2009), and tallied read counts across transcript positions to assess if ALT-ATGs exhibit read density patterns similar to 5p-ATGs that define the beginning of open reading frames (Methods). We observe qualitative similarity in read density profile between 5p-ATGs from all genes (fig. 3*a* top panel) and genes with ALT-ATGs (fig. 3*a* middle panel), in which 5p-ATGs have cumulative read sums with nearly an 8-fold difference from expected. For ALT-ATGs, we did not observe a similar fold change (fig. 3*a* bottom panel). Given the small number of genes included in our ALT-ATG analysis, we hypothesized that genes with high expression may skew our result. We therefore looked at position-specific read enrichment per gene (Methods). Again, we found a strong fold-change difference between the putative translation initiation site and surrounding positions (fig. 3*b*). In contrast, our gene-wise position-specific analysis revealed that ALT-ATGs have no difference in read support relative to surrounding positions.

We next assessed the lack of ribo-seq read density signatures more quantitatively by comparing read densities of our candidate ALT-ATGs against a randomly chosen INT-ATG from the same gene (comparisons with less than three normalized read counts combined were thrown out). We tested for differences in read support between ALT-ATGs and INT-ATGs from the same open reading frame. If ALT-ATGs do not represent true translation initiation sites, then the ALT-ATGs should have a higher read density than a randomly chosen INT-ATG 50% of the time. Validating this approach, 5p-ATGs have higher ribo-seq read density than a normal ATG 92.2% of the time (fig. 3*c*;*P* value ≤0.01 by a χ^2^ test), far higher than the expected 50%. By contrast, ribo-seq read densities at ALT-ATGs had higher read density only 55% of the time, which was not statistically different from the 50% expectation (*P* = 0.81 by a χ^2^ two-tailed test). Altogether, ribo-seq read support used in this study does not implicate ALT-ATGs as likely putative sites for translation initiation.

### Testing for Regulation of Pervasive Transcripts

If in fact, as the above results suggest, ALT-ATGs by and large do not represent functional alternative translation sites, this raises the question of how production of potentially harmful protein variants by translation of these ALT-TS isoforms is avoided. One mechanism of translation repression involves upstream ORFs (uORFs), in which the presence of a short ORF upstream of the TIS for the main ORF of a transcript leads to translational repression of the main ORF. Indeed, ribosomal profiling indicated that abundant short uORFs in *T. brucei* are occupied by the ribosome, suggesting roles in regulating gene expression (Vasquez et al. 2014).

**Fig. 4.**
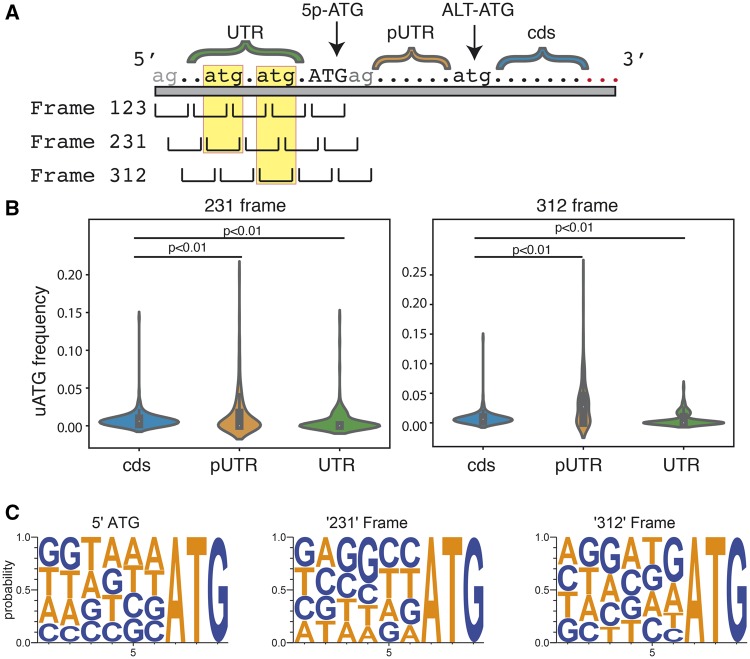
—uATGs are moderately enriched for in pUTRs. (*A*) Diagram showing different gene regions used in search for uATGs. UTR is defined as the region between the annotated TIS and any upstream SLTS acceptor site (gray “ag” dinucleotide). pUTR is defined as the region between internal SLTS acceptor site and downstream ALT-ATGs. CDS is defined as the region downstream from ALT-ATGs. (*B*) uATG frequencies for different sequence types for the 231 frame (left panel), and 312 frame (right panel). (*C*) WebLogo of uATG nucleotide context across reading frames.

In this case, a short ORF could disrupt translation from an ALT-ATG if it falls between the ALT-TS site and the ALT-ATG (fig. 4*a*). We therefore compared out-of-frame upstream AUGs (uATGs, for simplicity) within these intervening regions with normal protein-coding sequence to determine if the rate of uATGs is enriched in comparison to normal protein-coding sequences (fig. 4*b*). Overall, we found significant differences in uATG frequency between normal protein-coding sequence, UTRs, and ALT-TS-to-ATG regions (*P*≤0.01 by a Wilcoxon-rank sum test). Interestingly, when we divided AUGs into the two potential frames for out-of-frame ATGs (beginning on the second [“231”] or third [“312”] codon position, respectively), we found opposite patterns of relative uATG abundance in the two frames (fig. 4*b*): normal coding sequence has a higher density of uATGs in the 231 frame (*P *<* *0.01 by a Wilcoxon-rank sum test), whereas the ALT-TS-to-ATG regions have a nonsignificantly higher density of uATGs in the 312 frame. Despite the significant enrichment of uATG frequency within pUTRs relative to protein-coding sequence, the difference in magnitude is small (<5% difference from expectation in either the “312” or “231” frames).

## Discussion

Stimulated by the possibility that alternative trans-splicing might be a major generator of proteomic diversity (Benabdellah et al. 2007; Nilsson et al. 2010; Siegel et al. 2011; Rettig et al. 2012), we performed a multidisciplinary genome-wide study of potential alternative translation initiation sites. We specifically sought to determine the general functions of alternative trans-splicing by assaying for conservation of transcriptomic structure between two deeply diverged parasites. This approach is limited, in that it does not address lineage-specific functional ALT-TS. Although it is plausible that ALT-TS functions could be explained by host- or vector-related regulation for trypanosomes with varying developmental patterns, we did expect to observe conserved ancestral ALT-TS patterns, such as the case of isolecucyl-tRNA synthetase (Rettig et al. 2012). Nevertheless, we find two lines of evidence that ALT-TS is not a major mechanism driving proteomic diversification: potential ALT-TS-coupled alternative translation initiation sites do not show high ribosomal occupancy and are not preferentially evolutionary conserved.

Although these results suggest that ALT-TS does not greatly increase proteomic diversity on a genome-wide scale, individual cases of ALT-TS do lead to important alternative protein isoforms. Indeed, molecular and computational studies of isoleucyl-tRNA synthetase have shown that ALT-TS produces alternative mRNA isoforms that are targeted to different subcellular locations (Rettig et al. 2012). In our study, we do not find evidence that ALT-TS leading to dual localization of proteins is a widespread mechanism. In contrast, we find moderate levels of enrichment for out of frame ATGs, which suggests that ALT-TS occurring internal to open reading frames may produce degradation products. However, a thorough genetic and biochemical analysis is required to determine if such a mechanism is functionally conserved.

Relatedly, an interesting study in fungi may lend some insight as to how these trypanosomes regulate the expression of pervasive transcripts. Several studies have shown that a substantial number of transcripts in *Saccharomycescerevisiae* arise through the use of alternative promoters (internal to coding sequences), but the biological importance of such transcripts is not clear. In an elegant study, Malabat et al. (2015) showed that these pervasive transcripts, despite having downstream in-frame AUG-codons, are subject to degradation at the level of transcription and translation via exosome and nonsense-mediated decay. Though expression regulation in trypanosomes is widely speculated to be at the level of translation exclusively, it would be interesting to determine if ALT-ATG transcripts are subject to the same or similar regulatory pathways.

## Materials and Methods

### Read Mapping, SLTS Site Assignment, and ALT-ATG Calling

Spliced leader trapping data were obtained from European Nucleotide Archive (PRJEB4801) and mapped to *T.brucei* genome using bowtie2 with default parameters. Sequence alignment (BAM) files were converted to BED6 format using BEDTOOLS bamtobed, and intersected with *T.brucei* gene coordinates obtained from TriTrypDB annotations (release 5.0) using BEDTOOLS closest with -s option. Reads >2 kb away from the closest downstream gene were filtered out. SLT supported SLTS sites were then defined as major and minor, in which major sites contained the most SLT read support, and minor sites contained support of at least 10% of the major. Next, SLT reads were assigned to the first downstream ATG based on open reading frame annotations. Alternative-ATGs were defined as ATGs within an open reading frame with upstream SLT read support.

### Defining Orthologs and Computing Conservation of ALT-ATGs

A permutation-based approach was used to determine significant differences in conservation of 5p-ATGs and ALT-ATGs. To do this, we first generated an orthologous gene list for *T.brucei* and *T.cruzi* by aligning protein sequences for each open reading frame and defining best reciprocal hits using BLASTp. With these protein sequence alignments, for each ortholog, conservation was measured as the number of *T.brucei* ALT-ATG aligned with a corresponding ATG codon in *T.cruzi*, divided by total number of *T.brucei* ALT-ATGs. This same metric was used to compute conservation rate of ALT-TS AG acceptor sites, where additionally the AG conservation rates were measures in a frame-specific manner. Next, for each ALT-ATG containing gene, we randomly permuted across INT-ATGs and measured conservation rates using the same method, for 10^6 permutations. We defined the *P* values as the number of times INT-ATG conservation was observed at a level equal to or greater than the measured conservation of ALT-ATGs. Ribosome Profiling Data Handling. 

## Supplementary Material

Supplementary data are available at *Genome Biology and Evolution* online.

## Supplementary Material

evz217_Supplementary_Data
