# Supplementary figures and images for: On the Function of Trans-Splicing: No Evidence for Widespread Proteome Diversification in Trypanosomes
Source: Genome Biol Evol. 2019 Oct 10;11(10):3014–21. doi: 10.1093/gbe/evz217 (PMC6821157; doi:10.1093/gbe/evz217)

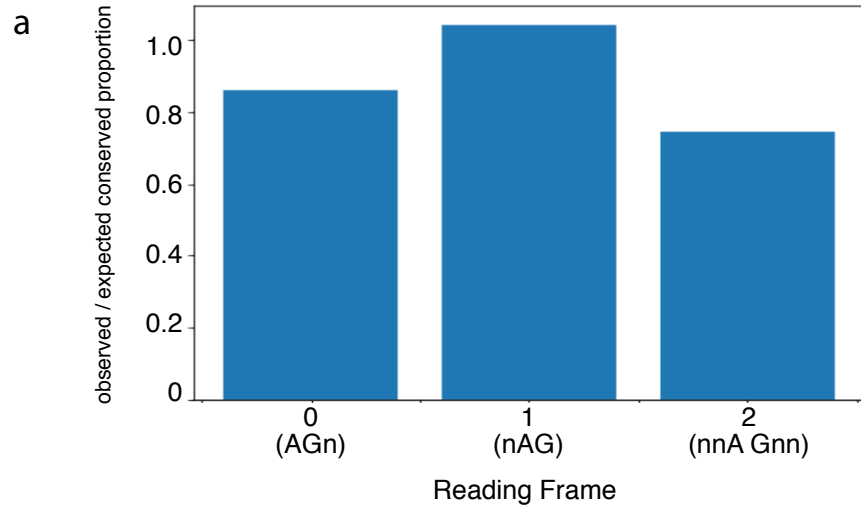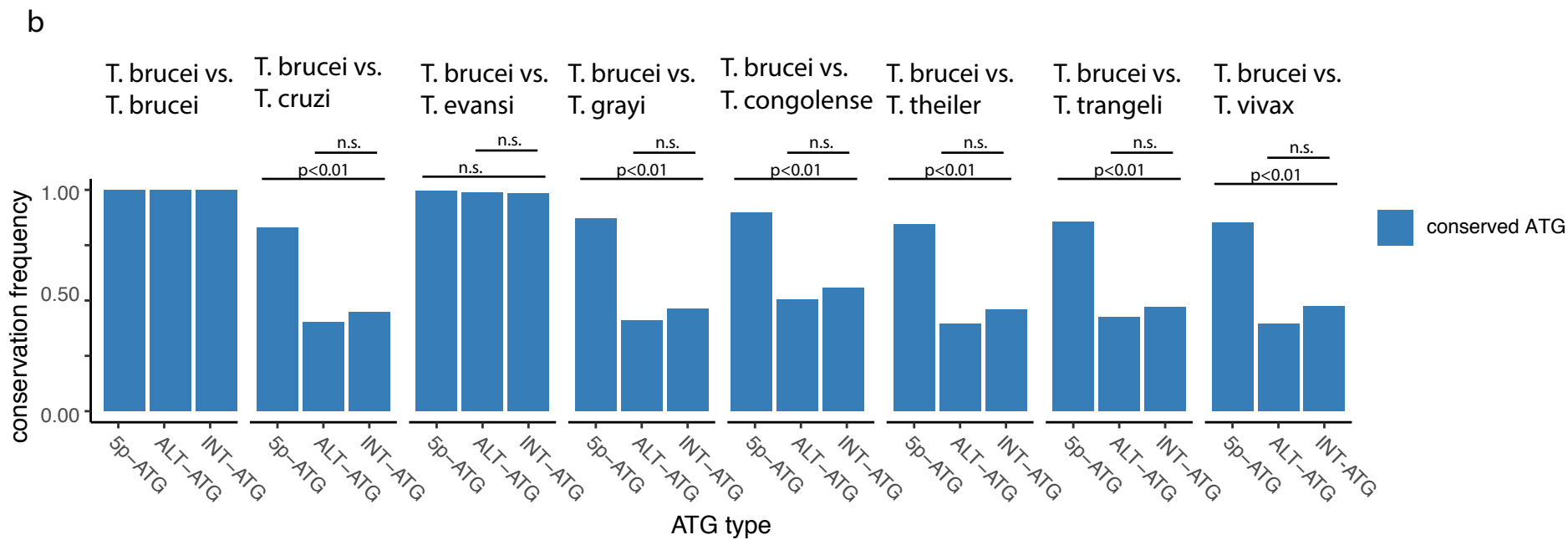

Supplement: evz217_Supplementary_Data [file evz217_supplementary_data.pdf]
